# Publisher Correction: Notch1 regulates the initiation of metastasis and self-renewal of Group 3 medulloblastoma

**DOI:** 10.1038/s41467-018-07182-1

**Published:** 2018-11-02

**Authors:** Suzana A. Kahn, Xin Wang, Ryan T. Nitta, Sharareh Gholamin, Johanna Theruvath, Gregor Hutter, Tej D. Azad, Lina Wadi, Sara Bolin, Vijay Ramaswamy, Rogelio Esparza, Kun-Wei Liu, Michael Edwards, Fredrik J. Swartling, Debashis Sahoo, Gordon Li, Robert J. Wechsler-Reya, Jüri Reimand, Yoon-Jae Cho, Michael D. Taylor, Irving L. Weissman, Siddhartha S. Mitra, Samuel H. Cheshier

**Affiliations:** 1 Division of Pediatric Neurosurgery, Lucile Packard Children’s Hospital, Stanford University School of Medicine, Stanford, 94305 California USA; 2 Institute for Stem Cell Biology and Regenerative Medicine and the Ludwig Cancer Center, Stanford University School of Medicine, Stanford, 94305 California USA; 3 Department of Neurosurgery, Stanford University School of Medicine, Stanford, 94305 California USA; 4 Ludwig Institute for Cancer Research, Stanford University School of Medicine, Stanford, 94305 California USA; 5 Division of Neurosurgery, Arthur and Sonia Labatt Brain Tumor Research Centre, Hospital for Sick Children, University of Toronto, Toronto, M5G 0A4 Ontario Canada; 6 Computational Biology Program, Ontario Institute for Cancer Research, Toronto, M5G 0A3 Ontario Canada; 7 Department of Immunology, Genetics and Pathology, Science for Life Laboratory, Rudbeck Laboratory, Uppsala University, Uppsala, 75185 Sweden; 8 Tumor Initiation and Maintenance Program, Sanford Burnham Prebys Medical Discovery Institute, 2880 Torrey Pines Scenic Drive, La Jolla, 92037 California USA; 9 Department of Pediatrics and Department of Computer Science and Engineering, University of California San Diego, San Diego, 92093 California USA; 10 Department of Medical Biophysics, University of Toronto, Toronto, M5G 1L7 Ontario Canada; 11 Department of Pediatrics, Children’s Hospital Colorado, University of Colorado, School of Medicine, Room No. P18-4114, Research Complex 1−North MS-8302, 12800 East 19th Avenue, Aurora, Colorado 80045 USA; 12 Division of Pediatric Neurosurgery, Department of Neurosurgery, Primary Children’s Hospital and Huntsman Cancer Institute, University of Utah, 100 North Mario Capecchi Drive Suite 3850, Salt Lake City, Utah 84113 USA

Correction to: *Nature Communications* 10.1038/s41467-018-06564-9; published online 08 October 2018

The original version of this Article omitted Suzana A. Kahn, Siddhartha S. Mitra & Samuel H. Cheshier as jointly supervising authors. This has now been corrected in both the PDF and HTML versions of the Article.

