# Supplementary material for: Notch1 regulates the initiation of metastasis and self-renewal of Group 3 medulloblastoma
Source: Nat Commun. 2018 Oct 8;9:4121. doi: 10.1038/s41467-018-06564-9 (PMC6175869; doi:10.1038/s41467-018-06564-9)
Supplement: Supplementary file 1 — Supplementary Information [file 41467_2018_6564_MOESM1_ESM.pdf]

Notch1 regulates the initiation of metastasis and self-renewal of Group 3 medulloblastoma

Kahn et al

Supplemetary Information

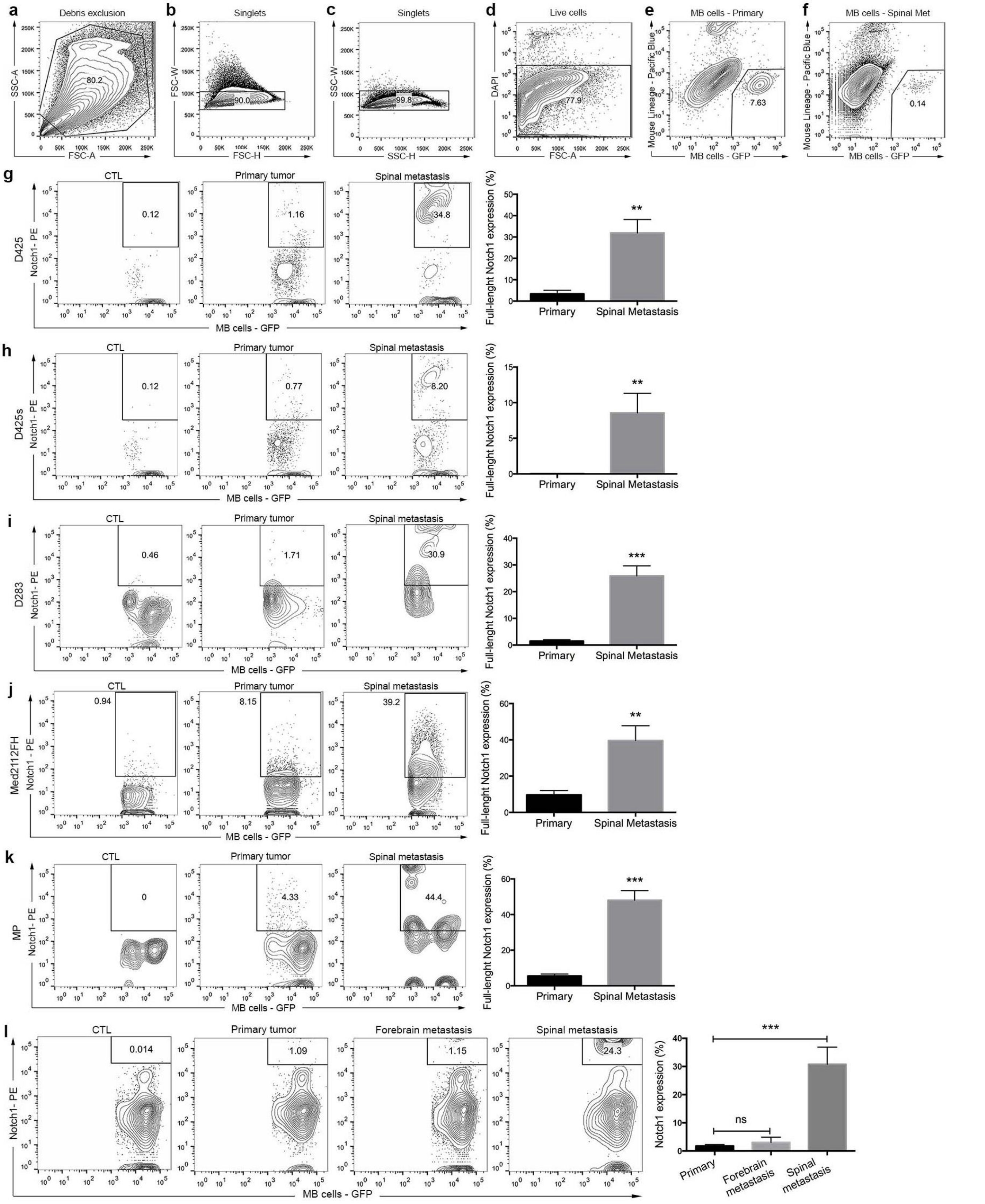

Supplementary Figure 1. Group 3 medulloblastoma cells isolated from spinal

metastases express higher levels of surface NOTCH1 than cells isolated from the primary tumors. a-f, Representative gating strategy to select and isolate human Group 3 medulloblastoma cells in primary tumors and spinal metastasis from xenografts. Flow cytometry gating were done for debris exclusion (**a**), single cell isolation (**b, c**), and purifying live population (**d**). Human cells are GFP^+ and mouse lineage cells are Pacific $Blue^+$. For medulloblastoma cells selection, GFP^+ / Pacific $Blue^-$ cells were isolated from the primary (**e**) and metastatic (**f**) sites. **g-k,** Flow cytometry analysis of surface NOTCH1 expression and quantification of three independent experiments in four human Group 3 medulloblastoma models: D425 (**g**), D425s (**h**), D283 (**i**), Med2112FH (**j**), and a mouse MYC-driven medulloblastoma model: MP (**k**). CTL, isotype control cells. ** $P < 0.01$, *** $P < 0.001$, Mann-Whitney U test. **l**, Flow cytometry analysis of surface NOTCH1 expression in group 3 medulloblastoma cells (D425) from primary tumors, forebrain metastases and spinal metastases, and quantification of three independent experiments. Error bars, s.d.

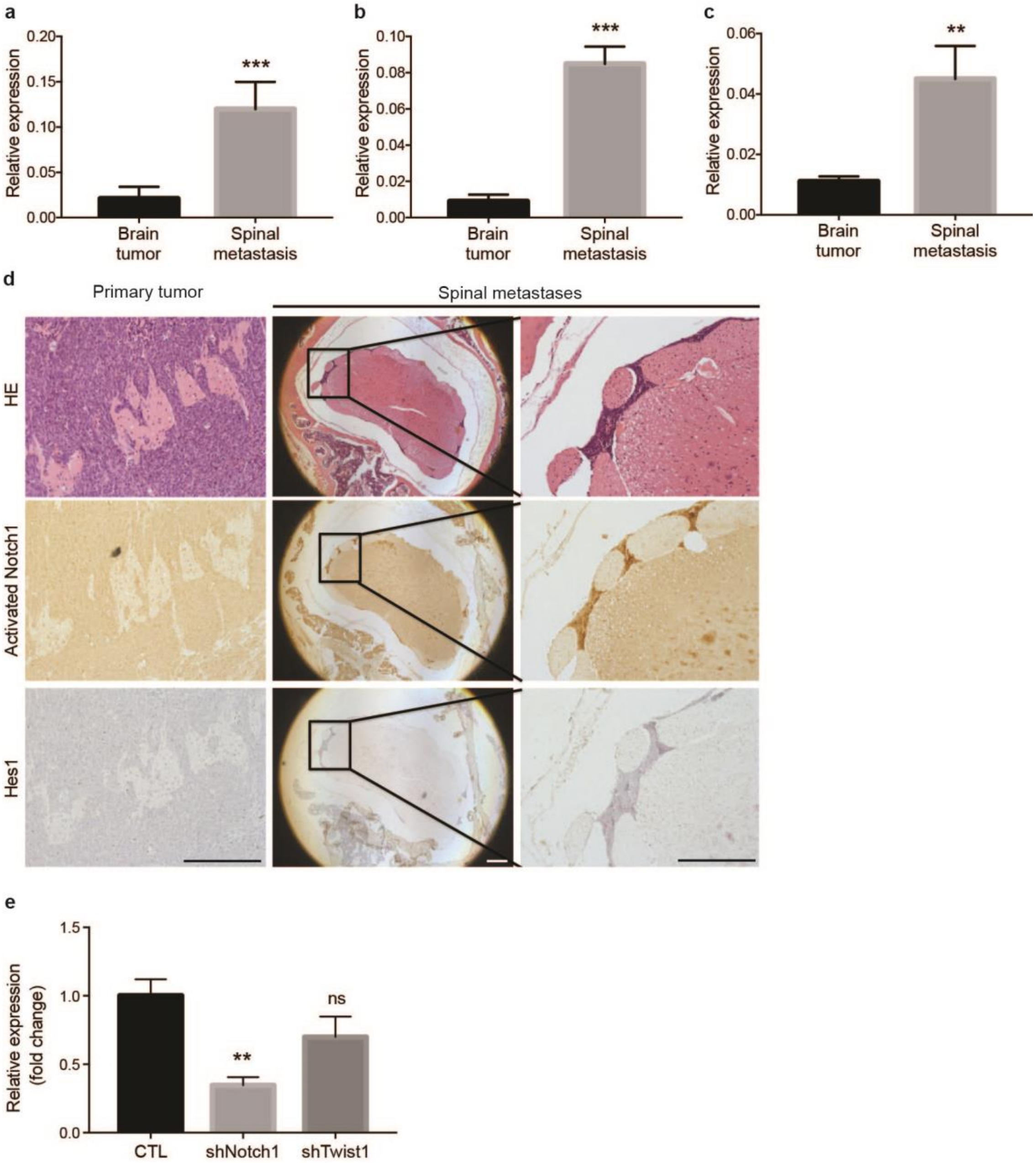

Supplementary Figure 2. Medulloblastoma spinal metastases express higher levels of activated Notch1 and Hes1 than primary tumors. a-c, qPCR for *HES1* in Group 3 medulloblastoma cells sorted from primary tumors and spinal metastases of mice injected with D425 (**a**), MB002 (**b**), and D283 (**c**). ** $P < 0.01$, *** $P < 0.001$, Mann-Whitney *U* test. **d**, Paraffin-embedded brain and spine from a MYCN-driven transgenic mouse [9]. Hematoxylin/eosin, activated Notch1 (DAB substrate), and Hes1 (VIP substrate) staining was performed in the primary tumor and spinal metastasis. Scale bars, 200μm. **e**, *NOTCH1*-silenced D425 cells express lower levels of *MYC* as compared to control. *TWIST1*-silenced D425 cells present similar levels of *MYC* expression as compared to control. ** $P < 0.01$, Mann-Whitney *U* test. Error bars, s.d.

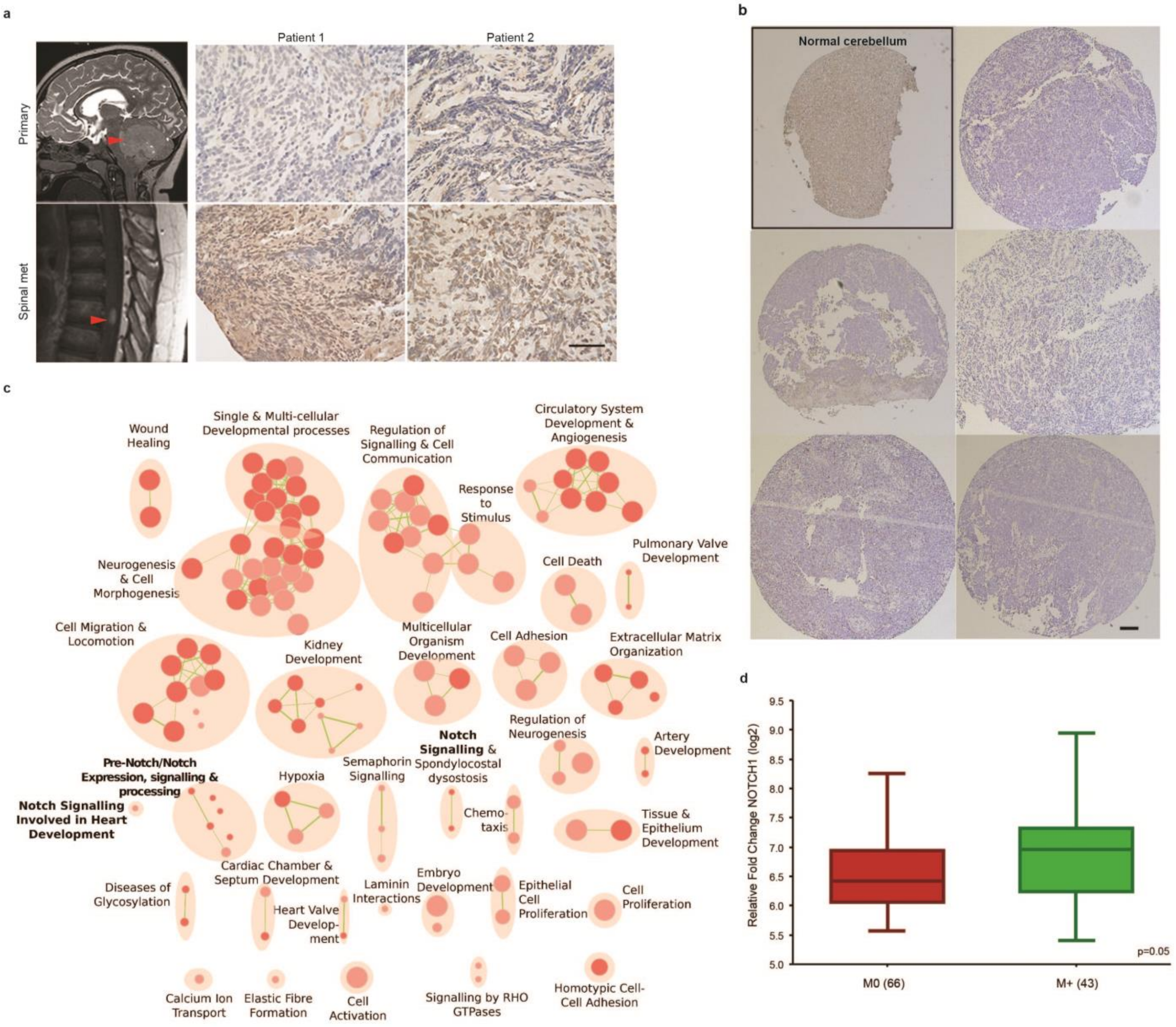

Supplementary Figure 3. NOTCH1 expression in human medulloblastoma. a, Representative MRI and immunohistochemistry for NOTCH1 in human Group 3 medulloblastoma from matched primary tumor and spinal metastasis samples from two patients. Spinal metastases samples show higher NOTCH1 expression (higher levels of DAB staining) compared to primary tumors from the same patients. Red arrowheads in the MRI images show primary and metastatic tumors. Scale bar, 100 μm. **b**, NOTCH1 is weakly expressed in human primary tumors. Normal human cerebellum was used as positive control. **c**, Enrichment map (see methods section) of significantly enriched pathways in *NOTCH1*-high primary tumors versus *NOTCH1*-low primary tumors (q<0.05 from g:Profiler [30]). NOTCH pathways are indicated in bold. **d**, *NOTCH1* expression is higher in Group 3 medulloblastoma patients that present metastasis (M+) as compared to patients that do not present metastasis (M0) at diagnosis. P = 0.05, Mann-Whitney U test. Error bars, s.d.

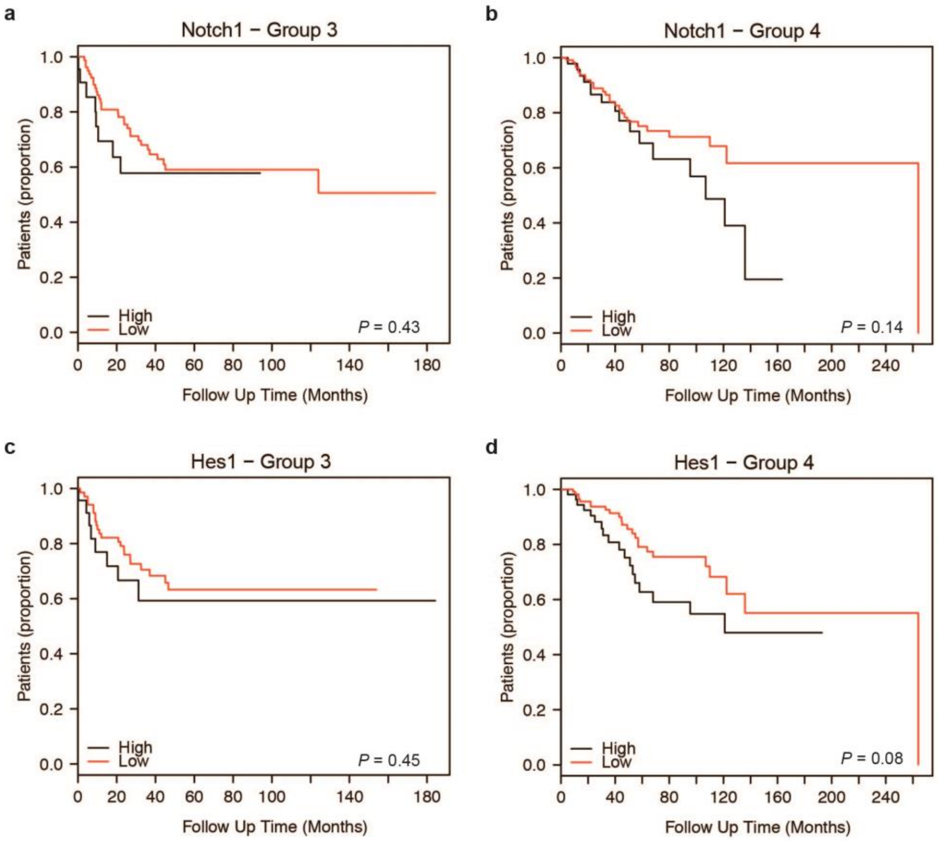

Supplementary Figure 4. *NOTCH1* and *HES1* expression correlate with the survival of medulloblastoma patients. **a**-**b** Survival curves of *NOTCH1*-low and *NOTCH1*-high expressing Group 3 (**a**) and Group 4 (**b**) medulloblastoma patients. **c**-**d**, Survival curves of *HES1*-low and *HES1*-high expressing Group 3 (**a**) and Group 4 (**b**) medulloblastoma patients. *P* values are from log-rank test.

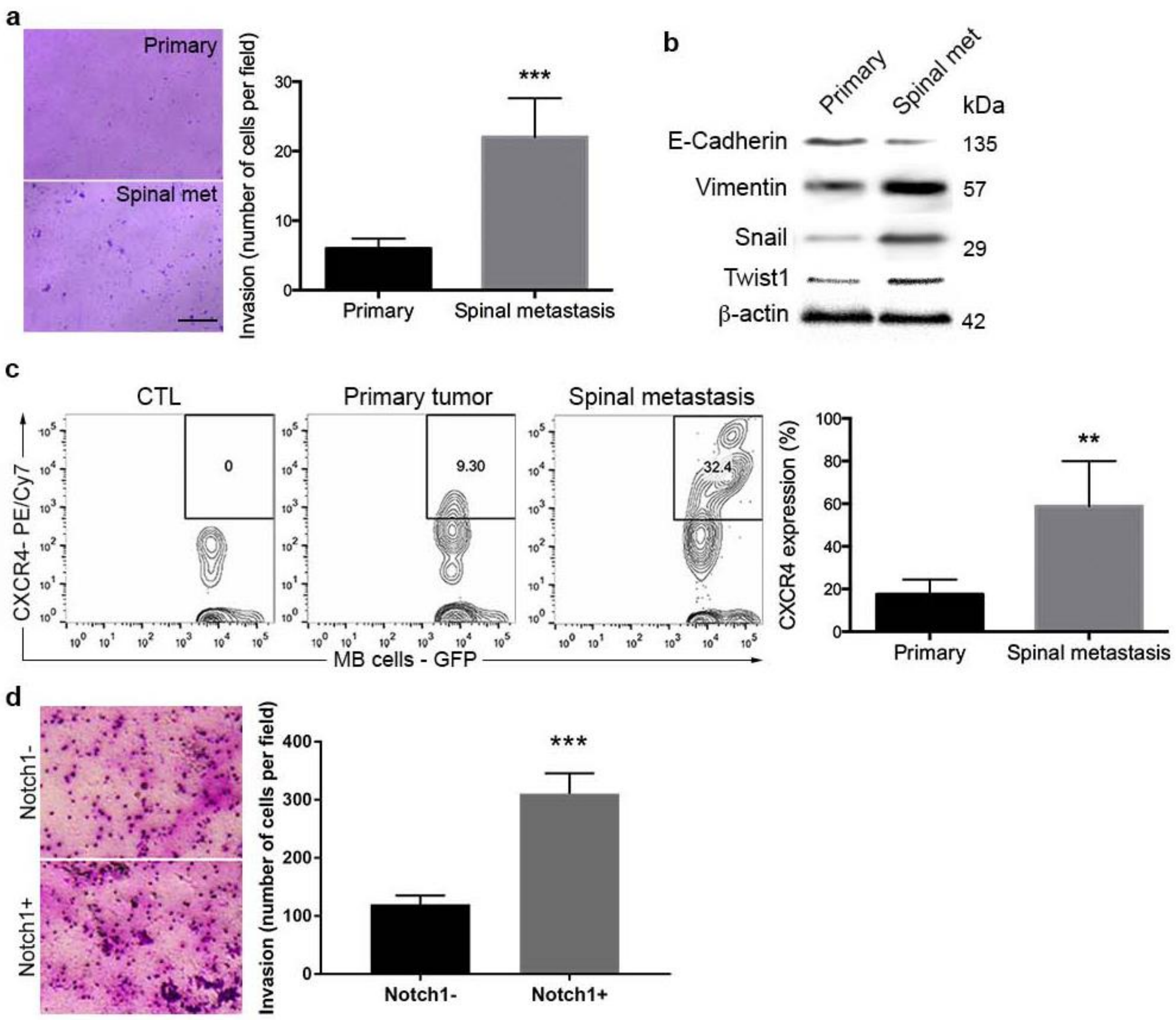

Supplementary Figure 5. Group 3 medulloblastoma cells from spinal metastasis are more invasive than medulloblastoma cells from the primary tumor site. a, Giemsa-stained Group 3 medulloblastoma cells (D425) isolated from primary tumor and spinal metastasis from xenografts after matrigel invasion assays and quantification of three independent experiments. *** $P < 0.001$, Mann-Whitney *U* test. **b**, Immunoblotting for E-cadherin (epithelial marker), Vimentin, Twist1 and Snail (mesechymal markers) in Group 3 medulloblastoma cells isolated from primary tumor or spinal metastasis from xenograft. kDa, Kilodaltons. **c**, Flow cytometry analysis of CXCR4 expression Group 3 medulloblastoma cells from primary tumors and spinal metastases from xenografts and quantification of three independent experiments. CTL, unstained cells. ** $P < 0.01$, Mann-Whitney *U* test test. **d**, Giemsa-stained NOTCH1+ and NOTCH1- Group 3 medulloblastoma cells (D425) after matrigel invasion assays and quantification of three independent experiments. *** $P < 0.001$, Mann-Whitney *U* test. Error bars, s.d.

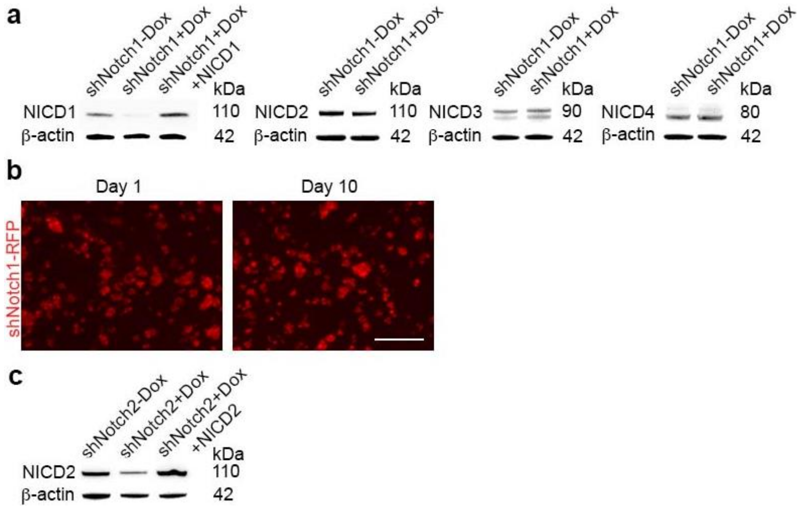

Supplementary Figure 6. NICD1 specific downregulation in Group 3 medulloblastoma cells. pTRIZ shRNAs specific to NOTCH1 (shNotch1) were introduced through lentiviral infections into Group 3 medullobalstoma cells (D425). Doxocyclin (Dox) was used to induce the expression of shRNA (shNotch1+Dox) and RFP. The uninduced infected cells (shNotch1-Dox) were used as controls. **a,** Immunoblotting for NICD1, NICD2, NICD3 and NICD4 in Group 3 medullobalstoma cells. kDa, Kilodaltons. β-actin was used as loading control. **b,** Fluorescence images of Group 3 medullobalstoma cells expressing the shNotch1-RFP after 1 day and 10 days of treatment with 2μg/mL of Dox. Scale bar, 200 μm. **c**, Immunoblotting for NICD2 in NOTCH2-silenced D425 cells (shNotch2+Dox), control (shNotch2-Dox) and NICD2 overexpression in NOTCH2-silenced D425 cells (shNotch2+Dox + NICD2). kDa, Kilodaltons. β-actin was used as loading control.

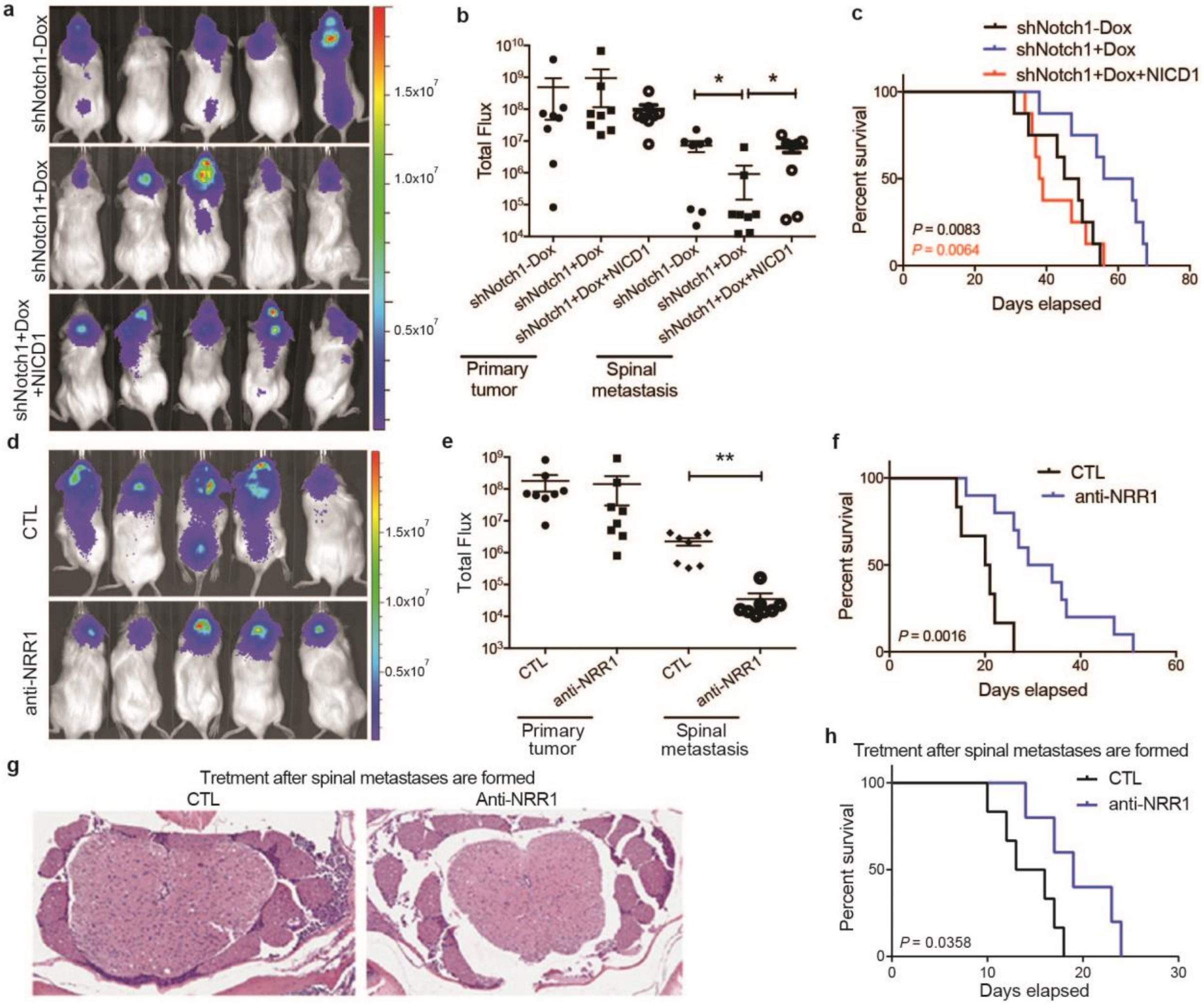

Supplementary Figure 7. Notch1 signaling regulates Group 3 medulloblastoma metastasis. a-**c**, shRNAs specific to NOTCH1 (shNotch1) were introduced through lentiviral infections into luciferase-expressing D283 cells. 2μg/mL of Dox was used to induce the expression of shRNA (shNotch1+Dox, blue). The un-induced infected cells (shNotch1-Dox, black) were used as controls. Recovery of NOTCH1 expression was performed by over-expressing NICD1 in NOTCH1-silenced Group 3 medulloblastoma cells (shNotch1+Dox + NICD1, red). Bioluminescence imaging (**a**) and quantification of total flux (**b**) from primary tumors and spinal metastases in mice injected with infected cells. * $P < 0.05$, Mann-Whitney *U* test. **c**, Kaplan-Meyer survival curves of mice injected with Group 3 medulloblastoma cells down-expressing NOTCH1 (shNotch1+Dox), control (shNotch1-Dox), or with recovered NICD1 expression (shNotch1+Dox + NICD1). *P* value is from log-rank test. **d**, Bioluminescence imaging of mice injected with luciferase-expressing MB002 cells and intrathecally treated with anti-NRR1 or vehicle (CTL). Quantification of total flux (**e**) quantification of total flux from primary tumors and spinal metastases. ** $P < 0.01$, Mann-Whitney *U* test. **f**, Kaplan-Meyer survival analysis of mice intrathecally treated with anti-NRR1 or vehicle (CTL). *P* value is from log-rank test. **g**, **h** hematoxylin/eosin staining of spines (g) and Kaplan-Meyer survival analysis (h) of mice intrathecally treated with anti-NRR1 after Group 3 medulloblastoma (MB002) spinal metastases were formed. Error bars, s.d.

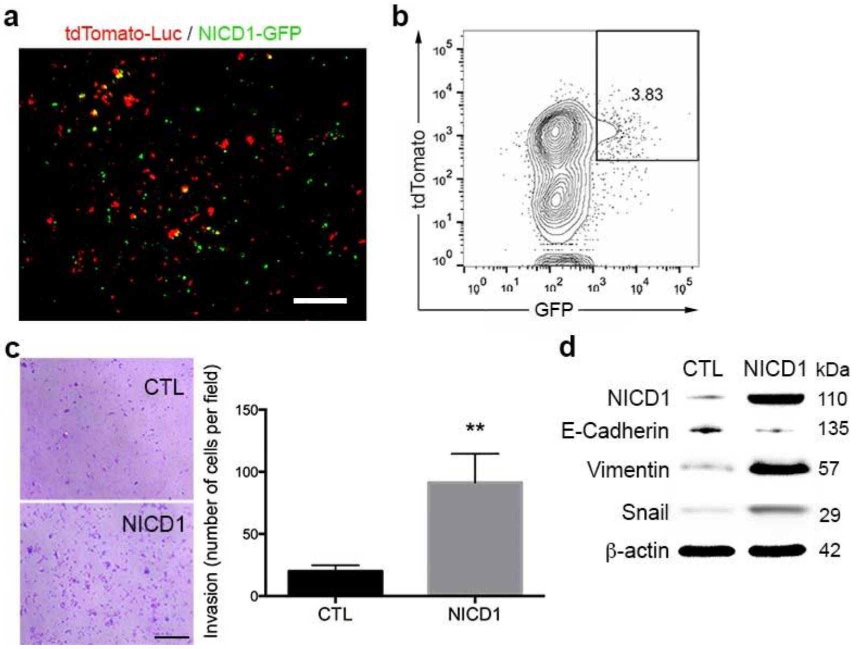

Supplementary Figure 8. NICD1 overexpression induces Group 3 medulloblastoma cells invasion *in vitro*. The constructs EF.hICN1.CMV.GFP (NICD1-GFP, green), for NICD1 overexpression, and Ubc.Luc.TdTomato (tdTomato-Luc, red) were introduced through lentiviral infections into Group 3 medulloblastoma cells. **a,** fluorescence image of infected medulloblastoma cells. **b,** Flow cytometry gating for selection of $tdTomato^+$ / GFP^+ medulloblastoma cells. Scale bar, 200 μm. **c**, Giemsa-stained Group 3 medulloblastoma cells overexpressing NICD1 and Control (CTL), after matrigel invasion assays and quantification of three independent experiments. ** $P < 0.01$, Mann-Whitney *U* test. **d**, Immunoblotting for NICD1, E-cadherin (epithelial marker), Vimentin and Snail (mesechymal markers) in Group 3 medulloblastoma cells transduced with EF.hICN1.CMV.GFP (NICD1) or CMV.GFP (CTL). kDa, Kilodaltons. Error bars, s.d.

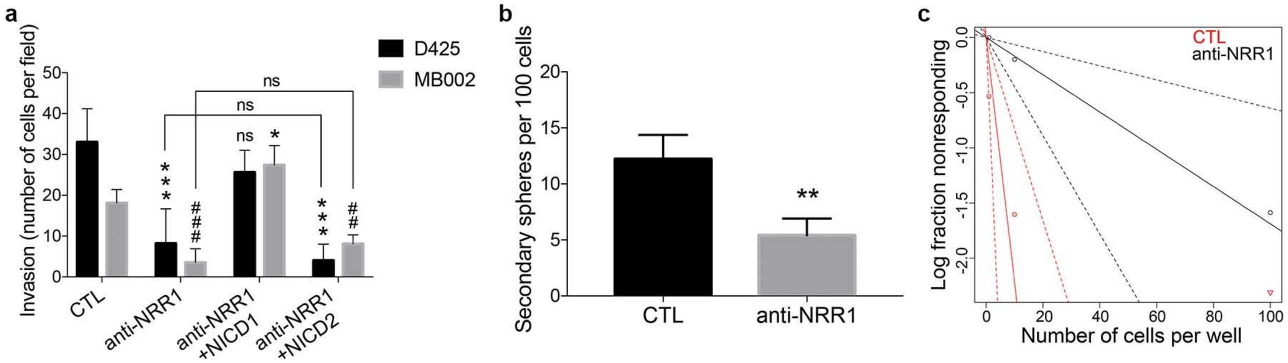

Supplementary Figure 9. Anti-NRR1 reduces invasion and neurosphere forming ability of Group 3 medulloblastoma cells *in vitro*. a, Quantification of Giemsa-stained medulloblastoma cells overexpressing NICD1 or NICD2 treated with anti-NRR1. NICD1 overexpression, but not NICD2, recovers the invasion ability of anti-NRR1-treated Group 3 medulloblastoma cells. * $P < 0.05$ versus D425 control; *** $P < 0.001$ versus D425 control; ## $P < 0.01$ versus MB002 control; ### $P < 0.001$ versus MB002 control, Mann-Whitney *U* test.
b, Quantitative analysis of the frequency of secondary colonies formed by Group 3 medulloblastoma cells treated with anti-NRR1 or control (CTL). ** $P < 0.01$, Mann-Whitney *U* test. **c**, *In vitro* extreme limiting dilution assays to single cells demonstrate that anti-NRR1-treated medulloblastoma cells present lower frequency of tumorsphere formation, as compared to control (CTL). Error bars, s.d.

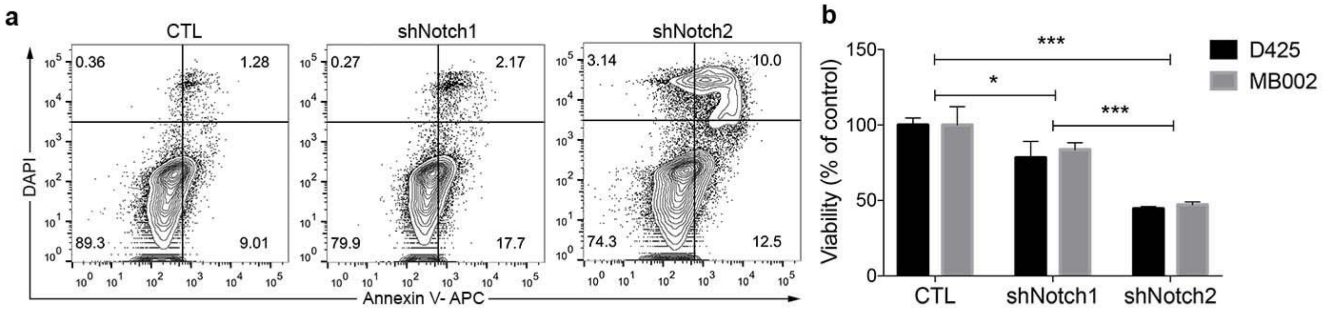

Supplementary Figure 10. NOTCH2 is involved in Group 3 medulloblastoma survival. a, Flow cytometry analysis of Annexin V and DAPI staining in NOTCH1-silenced and NOTCH2-silenced D425 cells. NOTCH2-silenced medulloblastoma cells present a higher percentage of Annexin V^+/ $DAPI^+$ cells, as compared to NOTCH1-silenced medulloblastoma cells. **b**, WST-1 proliferation analysis of NOTCH1-silenced and NOTCH2-silenced D425 and MB002 cells. NOTCH2-silenced medulloblastoma cells present lower viability than NOTCH1-silenced cells. * $P < 0.05$, *** $P < 0.001$, Mann-Whitney *U* test. Error bars, s.d.

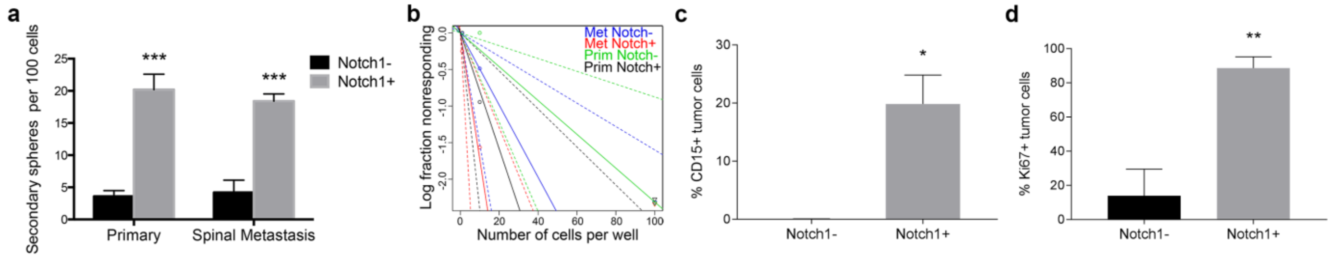

Supplementary Figure 11. NOTCH1$^+$ Group 3 medulloblastoma cells present higher neurosphere forming ability, CD15 and Ki67 expression than NOTCH1$^-$ Group 3 medulloblastoma cells. a, Quantitative analysis of the frequency of colonies formed by NOTCH1$^-$ and NOTCH1$^+$ Group 3 medulloblastoma cells isolated from primary tumors and spinal metastases from xenografts. *** $P < 0.001$ Mann-Whitney *U* test. **b,** *In vitro* extreme limiting dilution assays to single cells demonstrate that NOTCH1$^+$ Group 3 medulloblastoma cells from primary and metastatic tumors present higher frequency of tumorsphere formation than NOTCH1$^-$ tumor cells. **c**, Analysis of Group 3 medulloblastoma cells from the primary tumor site co-stained with NOTCH1 and CD15. Flow cytometry analysis of tumors from three mice. * $P < 0.05$, Mann-Whitney *U* test. **d**, Analysis of Group 3 medulloblastoma cells from the primary tumor site co-stained with NOTCH1 and Ki67. Flow cytometry analysis of tumors from three mice. ** $P < 0.01$, Mann-Whitney *U* test. Error bars, s.d.

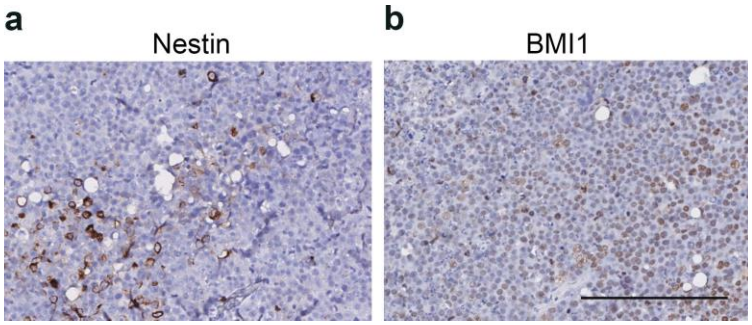

Supplementary Figure 12. Nestin and BMI1 expression in primary tumors from Group 3 medulloblastoma-bearing mice. a, b, Representative immunohistochemistry for Nestin (**a**) and BMI1 (**b**) of Group 3 medulloblastoma (D425) primary tumors. Scale bar, 200 μm.

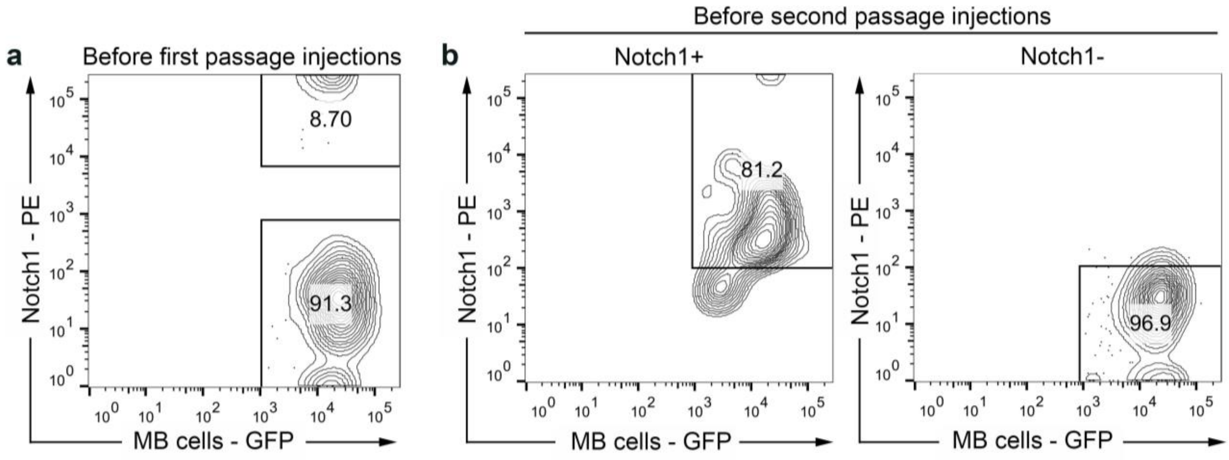

Supplementary Figure 13. NOTCH1⁻ and NOTCH1⁺ Group 3 medulloblastoma cells sorting for primary and secondary orthotopic transplantation. a, Flow cytometry plot showing $NOTCH1^+$ (8.7%) and $NOTCH1^-$ (91.3%) medulloblastoma cells gated for separation for the first *in vivo* passage. After dissociation of the primary tumors generated in mice, medulloblastoma cells were sorted based on surface NOTCH1 expression and reinjected into mouse cerebella (Figure 3d). **b**, Flow cytometry plots showing $NOTCH1^+$ (81.2%) and $NOTCH1^-$ (96.9%) medulloblastoma cells gated for separation for the second *in vivo* passage. After dissociation of the primary tumors generated by $NOTCH1^+$ and $NOTCH1^-$ Group 3 medulloblastoma cells, tumor cells were resorted based on surface NOTCH1 expression and reinjected into mouse cerebella (second *in vivo* passage).

Genes Over-Expressed in Spinal Metastasis	
**Gene Symbol**	**Fold Regulation**
ADAMTS1	51.3317
CX3CL1	33.7496
CXCL12	18.4288
CREBBP	15.1031
DTX1	21.6249
FOXC1	11.24
HES1	27.9225
HES5	12.2572
HEY1	25.1204
HEY2	8.2731
NODAL	6.2951
NOTCH1	8.9476
NOTCH3	6.4726
RBPJ	11.0092
RBPJL	7.724
SNAI1	20.5149
**Genes Under-Expressed in Spinal Metastasis**	
**Gene Symbol**	**Fold Regulation**
BDNF	-6.3226
CCN2	-10.5413
ID2	-8.4392
IL33	-15.4078
NAMPT	-6.5006
NRARP	-7.2361
SOX9	-11.8203
VEGFA	-17.2264

Supplementary Table 1. Expression of NOTCH pathway-associated genes in Group 3 medulloblastoma cells sorted from a primary tumor and its corresponding spinal metastasis. Fold change of genes over-expressed and under-expressed in spinal metastasis, as compared to primary tumor from xenograft.

Genes Over-Expressed in shNotch1	
**Gene Symbol**	**Fold Regulation**
ID3	10.3406
PCDH8	11.5323
RUNX1	6.4482
**Genes Under-Expressed in shNotch1**	
**Gene Symbol**	**Fold Regulation**
BDNF	-13.9607
CBFA2T3	-33.8289
CCND2	-22.7082
CLOCK	-117.6029
CREBBP	-849.9276
CX3CL1	-4.1571
CXCL2	-10.4438
DKK1	-21.5996
EDN1	-13.201
EFNA1	-5.8703
EFNB1	-5.2094
EGR3	-7.4524
FABP7	-11.2486
FJX1	-4.8937
FOXF1	-34.878
FRZB	-10.0136
GPSM2	-134.0143
HES1	-9.8682
HEY1	-999.8565
ID4	-6.0112
IGFBP3	-5.785
IL33	-11.5029
JUN	-703.3287
KITLG	-29.7286
KRT14	-9.28
MARK1	-1419.1911
NAMPT	-3513.6169
NES	-18.166
NODAL	-7.9165
NOTCH1	-15.2912
NOTCH3	-25.4164
PBX1	-111.2241
PDGFRB	-16.2111
PTGS2	-5.3739
S1PR3	-7.4246
SGPL1	-55.68
SOX9	-6.8491
WISP1	-13.6318
WNT6	-8.9571

Supplementary Table 2. Expression of NOTCH pathway-associated genes in Group 3 medulloblastoma cells sorted from primary tumor silenced for NOTCH1 expression. Fold change of genes over-expressed and under-expressed in primary tumors formed by NOTCH1-silenced medulloblastoma cells (shNotch1+Dox), as compared to primary tumors formed by control medulloblastoma cells (shNotch1-Dox).

Genes Over-expressed in anti-NRR1	
**Gene Symbol**	**Fold Regulation**
DKK1	104.6911
FJX1	10.5002
FOXD3	17.5492
FOXF1	125.0872
IGFBP3	88.1512
RUNX1	7.2842
SOX9	102.2254
WNT6	58.1226
**Genes Under-expressed in anti-NRR1**	
**Gene Symbol**	**Fold Regulation**
ADAMTS1	-69.2451
BDNF	-51.2367
CBFA2T3	-18.5129
CXCL1	-18.9013
CCND2	-69.6922
CLOCK	-20.6251
CREBBP	-31.3411
DTX1	-88.523
EDN1	-27.2887
EFNB1	-63.9801
EGR3	-45.1844
FABP7	-23.5271
FJX1	-89.9927
FOXC1	-49.2605
GPSM2	-7.5824
HEY1	-233.2675
HES1	-74.6526
HES5	-103.0041
ID4	-47.8105
KALRN	-5.2661
NES	-6.8176
NODAL	-21.6299
NOTCH1	-75.5445
NOTCH3	-48.4427
PDGFB	-15.0095
PDGFRA	-13.4825
PTCRA	-8.0703
PTGS2	-16.64
RBPJ	-128.6431
S1PR3	-11.2182
TCF15	-9.2469
WISP1	-27.4337

Supplementary Table 3. Expression of NOTCH pathway-associated genes in Group 3 medulloblastoma cells sorted from primary tumor from mice treated with anti-NRR1 or control. Fold change of genes over-expressed and under-expressed in medulloblastoma cells from primary tumors from mice treated with anti-NRR1, as compared to primary tumors from control mice.
